# Plasma Sphingomyelins and Carnitine Esters of Infants Consuming Whole Goat or Cow Milk-Based Infant Formulas or Human Milk

**DOI:** 10.1016/j.tjnut.2024.04.020

**Published:** 2024-04-12

**Authors:** Hans Demmelmair, Olaf Uhl, Shao J Zhou, Maria Makrides, Robert A Gibson, Colin Prosser, Sophie Gallier, Berthold Koletzko

**Affiliations:** 1 Department of Pediatrics, Division of Metabolic and Nutritional Medicine, Ludwig Maximilians University Munich, Dr. von Hauner Children’s Hospital, Munich, Germany; 2 Food and Wine, School of Agriculture, University of Adelaide, Adelaide, Australia; 3 Woman's and Children's Health Research Institute, University of Adelaide, Adelaide, Australia; 4 Science Department, Dairy Goat Co-operative (NZ) Ltd, Hamilton, New Zealand

**Keywords:** breastfeeding, infant formula, vegetable oil, goat milk, sphingomyelins, acylcarnitines

## Abstract

**Background:**

Infant formulas are typically manufactured using skimmed milk, whey proteins, and vegetable oils, which excludes milk fat globule membranes (MFGM). MFGM contains polar lipids, including sphingomyelin (SM).

**Objective:**

The objective of this study was comparison of infant plasma SM and acylcarnitine species between infants who are breastfed or receiving infant formulas with different fat sources.

**Methods:**

In this explorative study, we focused on SM and acylcarnitine species concentrations measured in plasma samples from the TIGGA study (ACTRN12608000047392), where infants were randomly assigned to receive either a cow milk-based infant formula (CIF) with vegetable oils only or a goat milk-based infant formula (GIF) with a goat milk fat (including MFGM) and vegetable oil mixture to the age ≥4 mo. Breastfed infants were followed as a reference group. Using tandem mass spectrometry, SM species in the study formulas and SM and acylcarnitine species in plasma samples collected at the age of 4 mo were analyzed.

**Results:**

Total SM concentrations (∼42 μmol/L) and patterns of SM species were similar in both formulas. The total plasma SM concentrations were not different between the formula groups but were 15 % (CIF) and 21% (GIF) lower in the formula groups than in the breastfed group. Between the formula groups, differences in SM species were statistically significant but small. Total carnitine and major (acyl) carnitine species were not different between the groups.

**Conclusions:**

The higher total SM concentration in breastfed than in formula-fed infants might be related to a higher SM content in human milk, differences in cholesterol metabolism, dietary fatty acid intake, or other factors not yet identified. SM and acylcarnitine species composition in plasma is not closely related to the formula fatty acid composition.

This trial was registered at Australian New Zealand Clinical Trials Registry as ACTRN12608000047392

## Introduction

Observational studies have associated breastfeeding with optimal infant growth, lower incidence of infections, lower long-term risk of obesity and type-2 diabetes, and potentially higher IQ-scores [1,2]. These findings strongly support the recommendation of exclusive breastfeeding for the first 4–6 mo of life [3]. Whenever breastfeeding is not possible, commercial infant formulas are a suitable alternative for infant feeding. The protein and carbohydrate components of formulas are typically based on cow skimmed milk and whey protein ingredients and the fat component on vegetable oils, resulting in structural and compositional differences to human milk fat, which may induce undesired nutritional consequences for the infant [4]. With a suitable mixture of vegetable oils and supplementation with long-chain polyunsaturated fatty acids, the fatty acid composition of infant formulas can be close to that of human milk [5]. However, the fatty acid complexity of milk fat and the components of the milk fat globule membrane (MFGM) present in human milk, and all animal milk, is missing. Instead, formulas include emulsifiers such as lecithin [6] and variable concentrations of polar lipids depending on the protein ingredients [7].

In addition to glycerophosphocholines (PC) and glycerophosphoethanolamines, a major class of MFGM polar lipids is sphingolipids, which can be differentiated into ceramides, phosphosphingolipids [sphingomyelins (SM)] with a phosphocholine head group, and neutral glycosphingolipids with glucose, lactose or more complex carbohydrate residues [8]. The MFGM contributes significant amounts of choline [9], cholesterol [10], and lipid-bound sialic acid from gangliosides [11] to the dietary intake of infants.

In contrast to MFGM, vegetable lecithin does not provide SM, and the proportion of saturated fatty acids in its glycerophospholipids is lower than in MFGM glycerophospholipids [7,12,13]. Comparisons between different formulas and human milk had previously focused on the total fatty acid composition, but with liquid chromatography-mass spectrometry, detailed comparisons of polar lipids, including SM species, have become available [5,14,15].

A few clinical studies point toward positive effects of MFGM intake on neurologic and immune system development of infants [[16], [17], [18], [19], [20], [21]]. However, there is little information on effects of MFGM intake on serum polar lipid species. The addition of MFGM is associated with higher serum cholesterol concentrations, closer to those found in breastfed infants, compared with standard formulas with only vegetable oils emulsified with soy lecithin [22]. Effects of different formulas or human milk on the global serum fatty acid composition have been reported, but SM species have hardly been studied [5]. In preterm infants, beneficial effects of SM supplementation in formula on neurobehavioral development have been observed [23]. Also, there is only limited information available on the effects of the fatty acid composition of infant formulas on serum concentrations of individual SM species. In 4-mo-old infants, Uhl et al. [24] reported significantly higher concentrations of several SM species in breastfed compared with formula-fed infants, but differences in the formula fatty acid composition were not reflected in the SM species. In the Cambridge Baby Growth Study, several shorter chain SM species were found at higher concentrations in breastfed infants at the age of 3 mo than in formula-fed infants, but total SM content was not significantly different [25]. In the Barwon Infant study differences of SM species between formula-fed and breastfed infants aged at 6 mo were small compared with differences of other studied lipid classes [26]. However, lipidomic analyses of serum and red blood cell membrane lipids from a Swedish study indicated that SM species contributed to the differentiation between infants fed formula supplemented with bovine MFGM compared with a control formula group [27].

Branched-chain amino acid catabolites and intermediates of fatty acid β-oxidation cross the mitochondrial membrane as carnitine esters, which make short-chain acylcarnitines indicators of amino acid catabolism and longer-chain acylcarnitines indicators of fatty acid oxidation intensity [28]. Although acylcarnitines are by now established markers for inborn errors of fatty acid oxidation, they are not frequently analyzed in infants as indicators of dietary effects on endogenous metabolism [29]. In a piglet model, feeding with goat milk induced higher serum triacylglycerol concentrations and differences in the mRNA expression of lipid metabolism-related genes compared with feeding human milk, cow milk, or infant formula [30]. Together with information about the fatty acid composition and the content of branched-chain amino acids in the diet, the analysis of carnitine species could support the understanding of protein and fat catabolism in infants and clarify if differences in fatty acid catabolism would lead to differences in the blood lipids in infants.

The Australian TIGGA study evaluated growth and nutritional status of infants fed a whole goat milk-based infant formula (GIF) containing milk fat and MFGM compared with a cow milk-based infant formula (CIF) without milk fat and MFGM. GIF was found to be well tolerated and provided equivalent infant growth compared with CIF [31]. Although the GIF and CIF were iso-caloric and matched as closely as possible for macronutrient content, the use of different fat sources resulted in differences in fatty acid composition, which may explain most of the differences previously reported in the infant plasma glycerophospholipids [32].

In the current study, we aimed to explore effects of these formulas on infant plasma SM and acylcarnitine species compared with a reference group of breastfed infants.

## Methods

### Subjects and study procedure

Plasma samples were obtained from infants participating in the TIGGA study, a multicenter, double-blind, controlled feeding trial in Australia (Australian New Zealand Clinical Trials Registry ACTRN12608000047392). Details on the study design and participating subjects were published [31]. Healthy term infants with a birth weight between 2.50 and 4.75 kg and age ≤2 wk were included and randomly assigned to receive either GIF or CIF (control) exclusively until at least 4 mo of age. Exclusively breastfed infants were enrolled as a nonrandomized reference group. The intervention formula GIF was manufactured using whole goat milk, and for the control formula CIF, cow skimmed milk, and whey proteins were used. Both formulas were provided by Dairy Goat Co-operative. Macronutrient composition of the formulas was very similar, but there were some differences in micronutrient contents [31]. The fat component of the GIF was a blend of 60% goat milk fat (and MFGM) and 40% vegetable oils, whereas the fat component of the control formula was a blend of vegetable oils emulsified with soy lecithin [33]. This resulted in a higher proportion of decanoic acid (7.3% compared with 2.1%) and a lower proportion of lauric acid (4.2% compared with 3.5%) in GIF [32]. Long-chain fatty acid percentages were similar between groups, but odd-chain heptadecanoic acid was 4 times higher in GIF than in CIF at 0.1% and 0.4%, respectively [32].

At 4 mo of age, plasma samples were obtained from 80% of the 301 recruited infants. In this study, we included 144 subjects (GIF = 57, CIF = 50, and human milk = 37). They were the subgroup with an available plasma aliquot for measuring SM and carnitine species.

### Sphingomyelin and carnitine species analysis

Targeted mass spectrometric analyses were performed at the Department of Pediatrics (LMU Munich, Germany) from 50 μL plasma as previously described [34]. Briefly, proteins were precipitated by adding 450 μL methanol containing as internal standards dimyristoyl-PC (Sigma), acetyl-L-carnitine-d3, octanoyl-L-carnitine-d3, and palmitoyl-Lcarnitine-d3 (Euriso-top). After centrifugation, the supernatant was further diluted with methanol and used for analysis of SM and carnitine species by flow injection-mass spectrometry with a 1200 SL HPLC system (Agilent) coupled to a 4000 QTRAP tandem mass spectrometer (AB Sciex). Positive ionization was applied and multiple reaction monitoring was used. Samples (6 technical replicates) of the study formulas were analyzed as described for the plasma samples after preparation according to the manufacturer’s instructions (14.0g/100 mL water).

Quantification, including background subtraction and isotopomer correction, was done using an inhouse programmed R script. Semiquantitative concentrations were obtained by comparing the signal-to-internal standard ratios of samples with the corresponding ratios of a control plasma (Recipe), whose SM and carnitine species were quantified using the Biocrates AbsoluteIDQ p150 Kit (Innsbruck).

The applied analytical technique is not capable of determining the positions of double bonds; thus, measured SM and acylcarnitine species were annotated based on the total number of carbon atoms and the total number of double bonds. For interpretation, the most likely number of carbon atoms in the sphingosine backbone and the N-acyl fatty acid was used. Aliquots of a mixture of plasma samples collected from healthy children were used as quality controls and consistently measured between study samples. Based on the measurement of 18 quality control aliquots, concentration data (μmol/L) for SM, free carnitine, and carnitine esters, where the coefficient of variation was <30%, were accepted and included in the data analysis.

### Statistics

Concentrations (μmol/L) are presented as mean and SD. Groups were compared by ANOVA, and post-hoc Bonferroni corrected comparisons of individual groups were performed. Due to multiple testing, the level of significance *P* < 0.05 was adapted for 20 SM species or 17 carnitine species, respectively. Correlation analyses were performed according to Pearson. A principal component analysis was carried out to visualize eventual separation of subjects according to the study groups. All statistical tests were performed with SPSS software version 26 (IBM).

## Results

Twenty-five SM species could be quantified (coefficient of variation ≤22%) in the GIF and CIF formulas (Table 1). Measured total SM content was similar (41.9 and 42.0 μmol/L in the GIF and CIF, respectively). The species concentration patterns were also similar, with the exception of SM39.1, which was 3 times higher in CIF.

**TABLE 1:** Concentrations of individual sphingomyelin (SM) species in ready-to-use cow milk-based (CIF) and whole goat milk-based (GIF) infant formulas (μmol/L) and percentage contributions of the 25 analyzed species to total SM.

	GIF (μmol/L)	GIF (%)	CIF (μmol/L)	CIF (%)
SM28:1	0.1	<1	ND^1^	<1
SM32:1	1.2	3	1.5	4
SM33:1	0.6	1	1.4	3
SM34:1	8.8	21	8.4	20
SM34:2	0.2	1	0.3	1
SM35:0	0.2	<1	0.2	<1
SM35:1	1.0	2	0.5	1
SM36:0	0.4	1	0.5	1
SM36:1	2.7	6	1.1	3
SM36:2	0.3	1	0.2	<1
SM37:1	0.5	1	0.3	1
SM38:1	1.0	2	2.1	5
SM38:2	0.2	1	0.2	1
SM39:1	1.3	3	4.3	10
SM39:2	0.2	<1	0.8	2
SM40:1	3.4	8	4.6	11
SM40:2	0.9	2	1.3	3
SM41:1	8.6	20	6.9	16
SM41:2	1.2	3	1.5	4
SM42:1	5.9	14	4.0	9
SM42:2	1.6	4	1.1	3
SM43:1	0.9	2	0.4	1
SM43:2	0.4	1	0.3	1
SM44:1	0.2	<1	0.1	<1
SM44:2	0.1	<1	0.1	<1
total SM	41.9		42.0	

1 Not detected

The plasma concentrations of most of the 20 SM species were higher in the breastfed group than in the formula groups, yielding significantly (*P* < 0.001) higher total SM in the breastfed group (296 ± 57 μmol/L) compared with 238 ± 41 μmol/L in the GIF group and 244 ± 46 μmol/L in the CIF group (Table 2). In the 2 formula groups, concentrations were similar, with significant group differences only for 4 SM species (Table 2). As the total of analyzed SM species was different between the breastfed and formula-fed groups, we also explored the percentage contribution of each species to total SM (Supplementary Table S1), which revealed a number of significant but small differences between groups. In all groups, the highest plasma concentration was found for SM34:1, followed by SM42:2. The contributions of SM40:2, SM38:1, and SM36:1 were rather similar in all groups.

**TABLE 2:** Plasma concentrations of sphingomyelin (SM) species (μmo/L, M±SD) of infants fed goat milk-based infant formula (GIF), cow milk-based infant formula (CIF), or human milk (HM); *P* values relate to Bonferroni corrected group comparisons post ANOVA considering multiple testing (20 species, ∗significance level *P* = 0.0025).

	GIF (*n* = 57)	CIF (*n* = 50)	HM (*n* = 37)	*P* value CIF-GIF	*P* value GIF - HM	*P* value CIF - HM
SM32:1	8.18 ± 2.50	12.32 ± 3.03	10.19 ± 2.68	3.5E-12∗	2.0E-03∗	1.4E-03∗
SM32:2	0.64 ± 0.28	0.48 ± 0.25	0.64 ± 0.31	9.4E-03	1.0E+00	2.6E-02
SM33:1	5.03 ± 1.25	5.14 ± 1.51	5.33 ± 1.61	1.0E+00	9.7E-01	1.0E+00
SM34:0	1.60 ± 0.61	1.47 ± 0.68	2.31 ± 0.61	8.3E-01	1.6E-06∗	3.1E-08∗
SM34:1	76.7 ± 14.6	75.3 ± 14.5	97.1 ± 22.4	1.0E+00	1.8E-07∗	6.4E-08∗
SM34:2	10.13 ± 1.99	9.16 ± 1.72	12.99 ± 3.29	9.9E-02	1.1E-07∗	1.1E-11∗
SM35:1	2.73 ± 0.69	1.55 ± 0.49	2.88 ± 1.04	4.7E-13∗	9.6E-01	2.6E-13∗
SM36:1	16.0 ± 3.6	18.3 ± 4.6	25.6 ± 7.2	7.2E-02	7.7E-15∗	2.1E-09∗
SM36:2	6.65 ± 1.45	4.69 ± 1.33	9.29 ± 3.00	1.9E-06∗	4.7E-09∗	4.1E-20∗
SM38:1	21.4 ± 5.9	26.3 ± 6.9	23.7 ± 6.4	3.5E-04∗	2.6E-01	1.9E-01
SM38:2	9.40 ± 2.63	9.79 ± 2.41	9.06 ± 2.18	1.0E+00	1.0E+00	5.2E-01
SM38:3	0.44 ± 0.19	0.40 ± 0.14	0.35 ± 0.12	6.1E-01	1.8E-02	3.5E-01
SM39:1	6.41 ± 2.23	7.87 ± 2.69	5.03 ± 1.95	4.6E-03	1.9E-02	3.4E-07∗
SM40:2	22.7 ± 7.1	24.0 ± 6.1	24.9 ± 5.6	9.1E-01	3.0E-01	1.0E+00
SM40:4	0.02 ± 0.01	0.02 ± 0.01	0.04 ± 0.01	1.0E+00	1.9E-12∗	3.5E-12∗
SM40:5	0.31 ± 0.18	0.25 ± 0.14	0.41 ± 0.20	1.3E-01	3.7E-02	9.6E-05∗
SM42:1	17.8 ± 4.8	14.7 ± 4.8	18.3 ± 4.4	2.9E-03	1.0E+00	1.5E-03∗
SM42:2	32.8 ± 7.9	32.4 ± 8.5	45.0 ± 12.5	1.0E+00	2.6E-08∗	2.4E-08∗
SM42:6	0.72 ± 0.59	0.78 ± 0.51	1.22 ± 0.74	1.0E+00	4.4E-04∗	3.2E-03
SM37:2	0.18 ± 0.10	0.20 ± 0.12	0.18 ± 0.10	7.9E-01	1.0E+00	1.0E+00
Total SM	238 ± 41	244 ± 46	296 ± 57	1.0E+00	4.7E-07∗	9.5E-06∗

**FIGURE 1:**
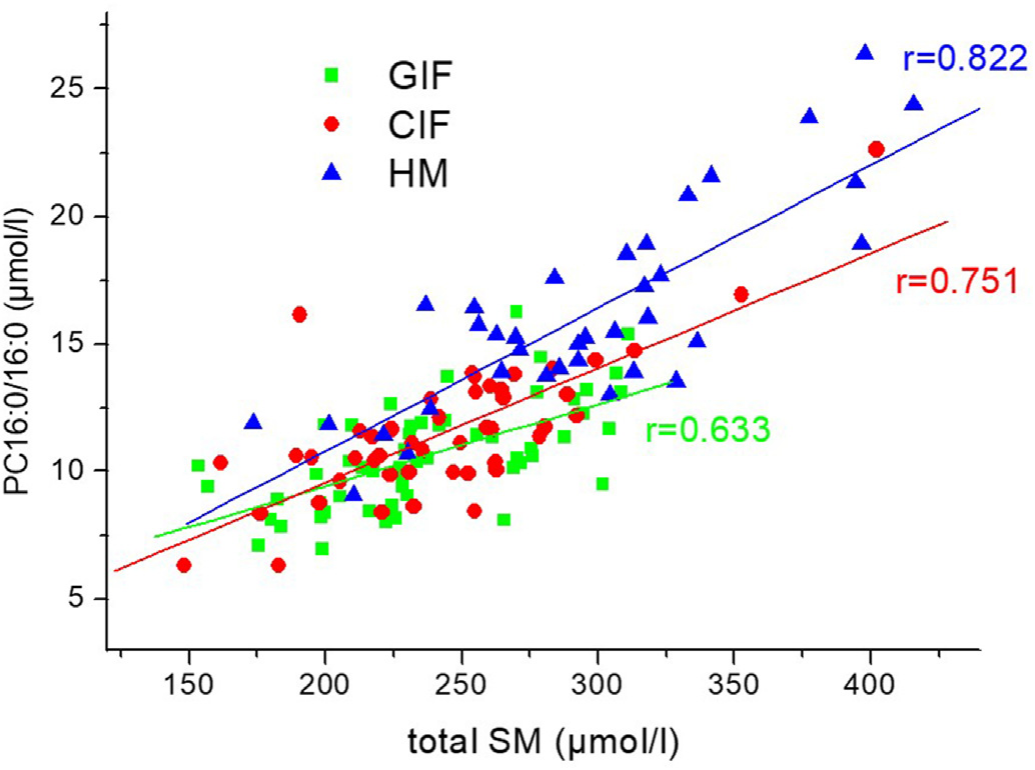
Association between the concentration of dipalmitoyl-glycerophosphocholine (PC16:0_16:0) and total sphingomyelin (SM) concentration stratified according to the study groups GIF (*n* = 55), CIF (*n* = 48) and HM (*n* = 36).

Effects of the different formula fatty acid compositions in the TIGGA study on plasma glycerophospholipid species have previously been published [32]. In the present study, we only investigated associations between SM and glycerophospholipids. Correlation analyses of plasma concentrations revealed that the sum of analyzed glycerophosphoethanolamines, Lyso-glycerophosphoethanolamines, Lyso-PC, and carnitine species was not correlated with total SM, but there was a highly significant association with the sum of the PC species (*r* = 0.622, *P* < 0.001). Of the 36 PC species quantified in ≥50% of the subjects, 23 were significantly associated with total SM with *r* > 0.5 in at least one of the study groups (Table 3) [32]. There was a clear trend toward closer correlations in the breastfed group than in the formula-fed groups. The r-values for PC species containing palmitic acid seemed higher than those of species with stearic acid (Table 3) [32]. Plasma PC16:0_16:0 showed the highest correlations with total SM in all study groups (Figure 1).

**TABLE 3:**
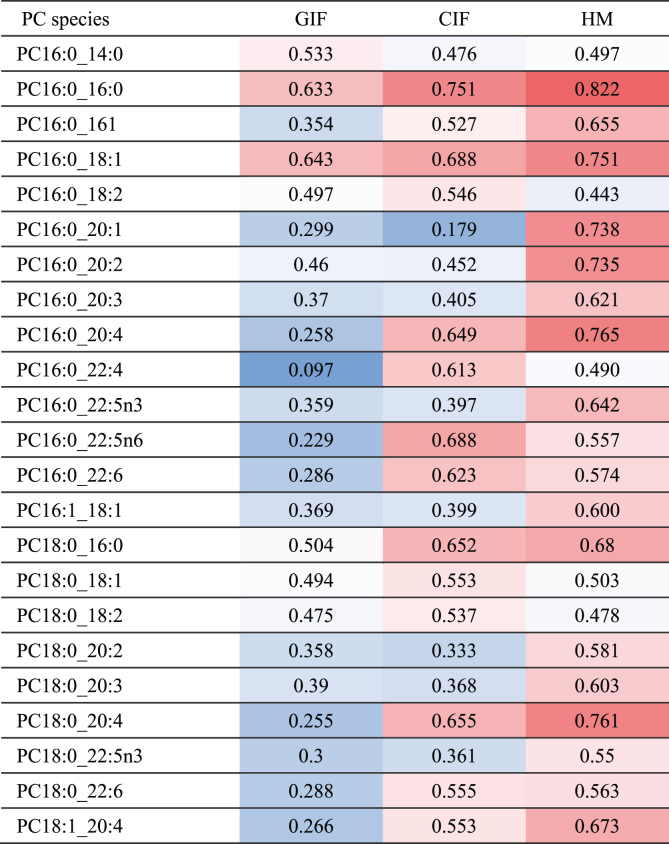
Pearson correlation coefficients (higher values: red, lower values: blue) between individual glycerophosphocholine (PC) species concentrations^1^ and total SM concentration stratified according to study groups.

1 Concentrations according to [32], determined by liquid chromatography-tandem mass spectrometry

**FIGURE 2:**
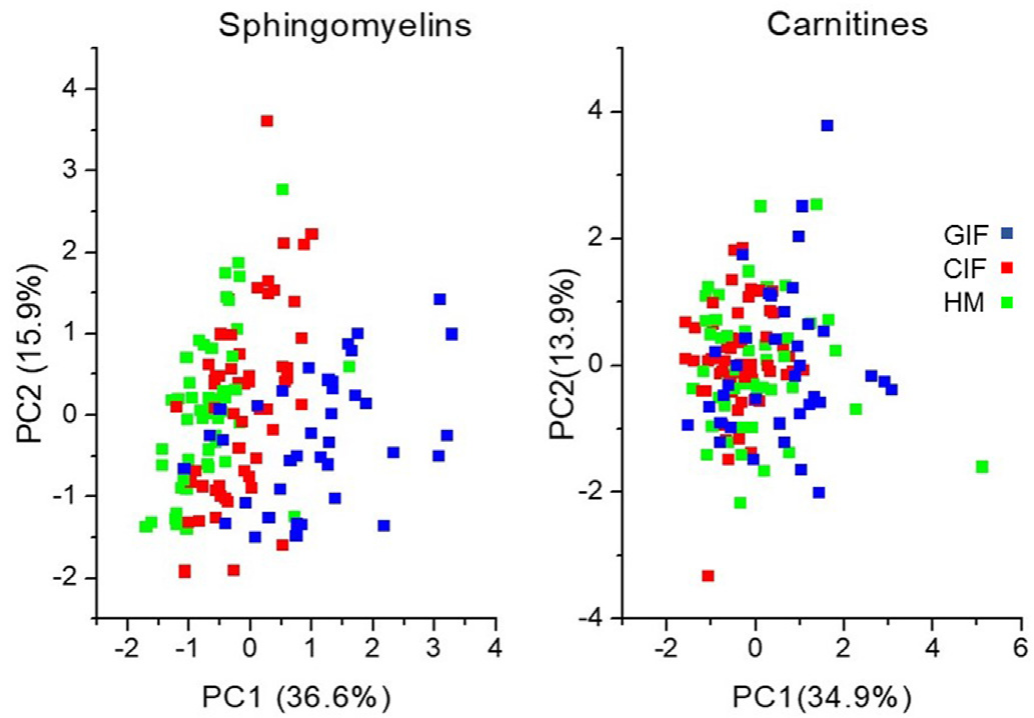
Score plot of principal components 1 and 2 for sphingomyelin species (left panel) and the carnitine species (right panel).

The 17 studied carnitine esters did not show a consistent picture, although there were some small but significant group differences in the concentrations of individual species (Table 4). Principle components of the measured carnitine species graphically indicated the similarity between the groups (Figure 2). In line with the concentrations, the plot of principal component 1 compared with principal component 2 for the SM species indicates some differentiation between the groups, with the GIF group being more similar to human milk than the CIF group (Figure 2, Table 2). SM and carnitine species analyses stratified for infant sex did not show different findings for males and females, respectively (data not shown).

**TABLE 4:** Plasma concentrations of carnitine species (μmol/L, M±SD) of infants fed goat milk-based infant formula (GIF), cow milk-based infant formula (CIF), or human milk (HM). *P* values relate to Bonferroni corrected group comparisons post ANOVA considering multiple testing (17 species, ∗significance level 0.003).

	GIF (*n* = 57)	CIF (*n* = 50)	HM (*n* = 37)	CIF vs. GIF	GIF vs. HM	CIF vs. HM
Free Carn	56 ± 10	56 ± 8	55 ±11	1.0E+00	1.0E+00	1.0E+00
Carn2:0	1.8 ± 0.9	2.2 ± 1.0	2.3 ± 1.1	1.9E-01	5.1E-02	1.0E+00
Carn3:0	0.30 ± 0.07	0.30 ± 0.10	0.34 ± 0.14	1.0E+00	2.5E-01	2.2E-01
Carn4:0	0.11 ± 0.04	0.11 ± 0.04	0.08 ± 0.02	1.0E+00	3.5E-04∗	1.7E-04∗
Carn5:0	0.23 ± 0.05	0.22 ± 0.06	0.21 ± 0.07	1.0E+00	3.4E-01	9.0E-01
Carn6:0-OH	0.04 ± 0.01	0.05 ± 0.02	0.04 ± 0.02	8.4E-01	1.0E+00	4.0E-01
Carn8:0	0.10 ± 0.03	0.12 ± 0.06	0.14 ± 0.05	3.4E-01	2.9E-04∗	4.2E-02
Carn8:0-OH	0.02 ± 0.01	0.02 ± 0.01	0.02 ± 0.01	1.0E+00	1.8E-01	6.8E-01
Carn8:1	0.27 ± 0.09	0.53 ± 0.19	0.21 ± 0.12	9.6E-17∗	1.2E-01	2.9E-19∗
Carn10:0	0.24 ± 0.08	0.25 ± 0.16	0.33 ± 0.15	1.0E+00	3.6E-03	1.3E-02
Carn10:1	0.10 ± 0.03	0.14 ± 0.04	0.13 ± 0.06	3.7E-06∗	2.4E-02	1.7E-01
Carn12:0	0.13 ± 0.04	0.17 ± 0.06	0.19 ± 0.06	1.2E-03∗	2.8E-06∗	2.5E-01
Carn12:1	0.07 ± 0.02	0.08 ± 0.04	0.10 ± 0.04	2.4E-01	1.9E-04∗	4.6E-02
Carn14:0	0.06 ± 0.02	0.06 ± 0.02	0.08 ± 0.03	1.0E+00	6.0E-02	1.5E-01
Carn14:1	0.06 ± 0.02	0.09 ± 0.04	0.07 ± 0.03	1.7E-05∗	4.7E-01	1.6E-02
Carn16:0	0.10 ± 0.05	0.11 ± 0.03	0.15 ± 0.05	1.0E+00	2.5E-05∗	4.3E-04∗
Carn18:1	0.14 ± 0.07	0.15 ± 0.06	0.15 ± 0.05	3.9E-01	9.6E-01	1.0E+00

## Discussion

The CIF and GIF formulas had been shown to be equivalent with respect to infant growth and wellbeing, but there were some biochemical differences, including higher plasma valine [31] and higher myristic acid and palmitoleic acid-containing PC species [32] in the GIF group. In the current analyses, we found small differences in the plasma concentrations of SM and carnitine species between the formula groups. Similar to the previous findings for PC species [32], there was a marked difference between formula-fed and breastfed groups with significantly higher total SM in breastfed infants.

Although the fat and emulsifier sources for both study formulas were different, total SM (42 μmol/L) was similar in the range reported recently for other infant formulas, which were mostly at lower concentrations than in human milk [[35], [36], [37]]. For GIF, the presence of SM is expected, as MFGM of whole goat milk was included in the formula [38]. The presence of SM in CIF is probably a residual from the whey protein ingredient used in the manufacture of CIF [39,40].

In both formulas, SM34:1, SM40:1, SM41:1, and SM 42:1 were among the 5 species with the highest concentrations and contributed together on a molar basis 64% (GIF) and 57% (CIF) to total SM. This is in agreement with findings in cow and goat milk [41]. Assuming that sphingosine is the dominant sphingoid base, this confirms that saturated fatty acids are preferentially incorporated into SM [39]. In agreement with Wei et al. [41], SM39:1 was higher in CIF than in GIF (10.2% compared with 3.2%), although other SM species, probably also including odd-chain fatty acids, were not different between formulas.

Our observation that total plasma SM is higher in breastfed infants than in formula-fed infants does not agree with previous findings based on the analysis of blood spots collected at the age of 3 mo in the Cambridge Birth Cohort Study [25]. However, similar findings were reported in the BEMIM trial, where about half of the SM species quantified in plasma were higher in the breastfed than in the formula-fed groups [24]. Furthermore, in an Australian cohort at the age of 6 mo, total serum SM was found to be significantly higher in breastfed than in formula-fed infants [26]. We speculate that SM concentrations in lipoproteins differ more between formula-fed and breastfed infants than SM incorporated into red blood cells.

The intestinal activity of sphingomyelinases and ceramidases releases absorbable sphingosine from dietary SM [42]. After absorption, only a portion of the sphingosine is recycled into ceramides and SM, whereas a major part is broken down to palmitic acid and ethanolamine in intestinal cells [43,44]. Thus, increased sphingosine availability may only have a limited effect on systemic SM synthesis. However, in preterm infants significantly higher contributions of SM to total plasma phospholipids have been observed in infants receiving a formula with higher SM content [23]. Thus, this suggests that higher SM intake can increase SM concentrations in the circulation. In addition, higher cholesterol intake and blood concentrations, including higher LDL:HDL cholesterol ratio, in breastfed infants compared with formula-fed infants [[45], [46], [47]] might contribute to the higher SM in the breastfed group, as suggested in observational studies in older adults where all measured SM species were significantly positively associated with total cholesterol, and SM percentage of total lipids was higher in LDL than in HDL [[48], [49], [50]].

In our study, the observed SM species concentration differences between formula groups were not related to the small percentage differences of myristic, palmitic, stearic, and oleic acid between GIF and CIF. The C17:0 content was 4 times higher in GIF than in CIF formula, which agrees with the typically higher odd-chain fatty acid contents in ruminant-derived fat compared with vegetable oils [51] and compared with CIF, the GIF group had a higher plasma content and percentage of SM 35:1, which can plausibly be annotated as SMd18:1/17:0 or SMd17:1/18:0. Serine palmitoyl transferase accepts fatty acids with chain length of 14–18 C-atoms as substrates [52] and heptadecanoic acid falls well into the substrate spectrum of ceramide synthases 4 to 6 [53]. Thus, SM35:1 could be generated via both routes. Relative to the small C17:0 content of the formulas (0.1% and 0.4% for CIF and GIF, respectively), the difference in the contents seems relevant and could become visible in the SM pattern.

A lipidomic study exploring biomarkers highlights the potential importance of endogenous metabolism and early life programming [54]. Concentrations of SM39:1 could differentiate control infants from infants born small for gestational age or born to mothers who developed gestational diabetes, whereas there were no differences between breastfed, formula-fed, or mixed-fed infants [54].

High-fat diets increased total SM and ceramides concentrations in animal studies, but the increase was significantly higher with palmitate compared with medium-chain fatty acids [55,56]. Furthermore, an SM-lowering effect of an olive oil diet compared with a coconut oil diet was observed in rats [57]. This is in line with our finding of a high correlation between total SM and dipalmitoyl-PC (Figure 1). Furthermore, this may contribute to the similar total SM in both formula groups, as the weight percentages of short and medium-chain fatty acids (C4 to C14) were very similar in both groups, with ∼19%, whereas it has been found lower ∼14% in Australian human milk [58]. The palmitic acid percentage of 22.3% in Australian milk reported by Yuhas et al. [58] is only slightly higher than the percentages in the study formulas (GIF: 17.4%, CIF: 21.7%). Nevertheless, considering that in human milk, in contrast to formula, the majority of palmitic acid is sn-2 bound and better absorbed, this might as well contribute to the higher SM concentration in the breastfed group than in formula infants [59].

A prominent role of palmitic acid appears not to be surprising, as palmitic acid is combined, in the initial step of SM synthesis, with serine to form 3-ketosphinganine, and palmitoyl-CoA is used as substrate fatty acid for the N-acylation of dihydrosphinganine by ceramide synthases 5 and 6 [53]. Of note, the so far described 6 ceramide synthases have different substrate preferences and well-documented tissue-specific expression [53]. Although some ceramide synthases transfer very long-chain fatty acids (C22-C26), others have preferences for shorter acyl chains from 14–20 carbon atoms [53]. The important role of ceramide synthases in SM species composition is supported by the observation in rats that fatty acid infusion increased total SM species composition, which was not determined by the composition of the infused fatty acids [60].

The acyl chain length of ceramides seems important regarding cardiovascular disease risk, with the shorter chain ceramides showing more detrimental effects [61,62]. Such observations have so far not been reported for SM species, but similarities of the SM and ceramide species patterns can be expected, considering that SM are metabolically closely interlinked with ceramides. Ceramides are intermediates in SM synthesis, and the larger SM pool acts as a precursor pool for ceramides if sphingomyelinase converts SM to ceramides [55].

The observed concentrations of free carnitine and acylcarnitines were in the range found in 3–4-mo-old infants in a recent German study [63]. Similar to the group differences in the SM species, corresponding differences among plasma carnitine esters were not closely related to differences in the fatty acid composition of the study formulas. Although carnitine content was higher in CIF compared with GIF (3.3 compared with 1.2 mg/100 kcal), plasma free carnitine and acetyl-carnitine, which contributed together 95% or more to total measured carnitine species, were not different between the groups. Although carnitine concentrations can be influenced by diet, intake differences may be attenuated by endogenous synthesis. Because the ratio of free carnitine to total carnitines is clearly >0.7 in all groups, there are no indications of carnitine deficiency in any group [64]. Only the higher lauric acid percentage in CIF (about 3 times higher than in GIF) was reflected in a significantly higher lauric acid carnitine ester content, whereas other smaller differences in the formula fatty acid composition did not seem to influence acylcarnitine concentrations. Low correlations between serum phospholipid long-chain fatty acids and acylcarnitines, as observed in an adult cohort, agree with the assumption of a limited influence of diet on carnitine species, as it is known that dietary fatty acids are reflected in serum phospholipids [65].

Serum concentrations of acylcarnitines with ≥ 6 carbon atoms are assumed to represent intermediates of the fatty acid beta-oxidation, thus reflecting mitochondrial processes rather than substrate availability [66,67]. Beta-oxidation of fatty acids does not seem much different between the study groups, although there seems to be a trend toward higher concentrations of medium and longer-chain acylcarnitines in breastfed infants.

It is important to note that differences in branched-chain amino acid intake not only change plasma amino acids but also the concentrations of the carnitine esters of their C3, C4, and C5 breakdown products [68]. In the TIGGA study, the feeding of formulas and human milk induced only small differences in plasma concentrations of leucine and isoleucine, respectively, but in both formula groups, plasma valine concentration was significantly higher than in the breastfed group [31]. The valine difference was reflected by significantly higher C4-carnitine, which includes the carnitine ester of the valine catabolite isobutyric acid, in the formula groups compared with the breastfed group. In our study, the serum concentration of octanoyl carnitine (Carn 8:1) was about twice as high in the CIF group than in the GIF group, although both its precursor in the beta-oxidation and its hydration product generated in the next step of the cycle (Carn8:0-OH) were not different between the groups. We cannot identify a mechanism to explain this finding, which might be a chance finding, also considering the unclear identity of the signal annotated as C8:1 carnitine [69].

In a Swedish study comparing formulas with or without MFGM with a breastfed reference group, plasma samples were collected at the age of 6 mo for metabolomics [70]. The study found higher concentrations of some long-chain acylcarnitines and ketones but lower short-chain acylcarnitines in the breastfed infants than in both formula-fed groups. This was interpreted as an indication of more fat oxidation in breastfed infants, whereas formula-fed infants showed more protein catabolism [70]. Although only statistically significant for palmitoyl-carnitine and butyryl-carnitine, a corresponding trend could be observed in the TIGGA study.

Other extensive analyses of carnitine and SM species in breastfed and formula-fed infants have been reported [26], but to our knowledge, this is one of the first studies quantifying individual SM plasma concentrations in infants in relation to mode of feeding. A high number of SM species could be quantified with our method, and the only assumed major missing species was SM40:1, the docosanoic acid-containing SM species. Nevertheless, there are some limitations to our study. The conclusions drawn from this study are based on the comparison of human milk and formulas produced from different raw materials. The various compositional differences preclude a definitive allocation of findings to a specific compound. Furthermore, only plasma samples were analyzed, and conclusions might have been different if red cells or even tissue samples had been available for analysis. The interpretation would have benefited from a more detailed knowledge of the polar lipids of the infant formulas and even more from the availability of human milk samples from the breastfed group. We could not identify all SM and carnitine species precisely because the positions of double bonds could not be located, but this could widely be overcome by valid assumptions based on pre-existing knowledge.

In conclusion, we show that total SM plasma concentration is higher in breastfed than in formula-fed infants, which might be related to higher SM concentration in human milk compared with the study formulas, but differences in cholesterol metabolism and lipoproteins between breastfed and formula-fed infants and further not identified factors may contribute as well. The species composition of plasma SM and acylcarnitines is not closely related to the dietary fatty acid composition or the addition of MFGM lipids to formula. We identified significant positive correlations between saturated PC species and the total SM plasma concentration, which might indicate options for modulating serum and potentially tissue SM contents. Human milk, CIF, or GIF feeding did not induce differences between plasma SM and carnitine species patterns that would indicate major differences in sphingolipid metabolism or fatty acid oxidation. Further studies should explore associations between SM and ceramide species, which are considered important risk factors for atherosclerotic disorders [71].

## Data Availability

Data described in the manuscript, code book, and analytic code will be made available upon request pending application approval and permitted by applicable rules of personal data protection.
