# Supplementary material for: Plasma Sphingomyelins and Carnitine Esters of Infants Consuming Whole Goat or Cow Milk-Based Infant Formulas or Human Milk
Source: J Nutr. 2024 Apr 12;154(6):1781–9. doi: 10.1016/j.tjnut.2024.04.020 (PMC11217027; doi:10.1016/j.tjnut.2024.04.020)
Supplement: Multimedia component 1 [file mmc1.docx]

Demmelmair et al: Plasma sphingomyelins and carnitine esters of infants consuming whole goat or cow milk-based infant formulas or human milk

**Supplementary Table 1:** Percentages (M±SD) of individual SM species in the plasma samples of infants fed CIF, GIF or HM; p-values below 0.0025 are considered significant and printed in bold

|  | GIF | CIF | HM | p-value  CIF-GIF | p-value  GIF - HM | p-value  CIF - HM |
| --- | --- | --- | --- | --- | --- | --- |
| SM32:1 | 3.39±0.76 | 5.08±0.79 | 3.50±0.86 | **2.5E-19** | 1.0E+00 | **6.0E-15** |
| SM32:2 | 0.27±0.10 | 0.20±0.10 | 0.22±0.10 | **2.2E-03** | 7.6E-02 | 1.0E+00 |
| SM33:1 | 0.67±0.24 | 0.60±0.26 | 0.80±0.25 | 1.0E+00 | 7.7E-03 | 1.3E-02 |
| SM34:0 | 2.11±0.40 | 2.10±0.54 | 1.81±0.41 | 4.0E-01 | 4.2E-02 | **7.3E-04** |
| SM34:1 | 32.19±3.97 | 30.87±2.78 | 33.03±3.04 | 1.4E-01 | 7.5E-01 | 1.2E-02 |
| SM34:2 | 4.28±0.70 | 3.76±0.51 | 4.43±0.64 | **1.5E-04** | 7.3E-01 | **8.0E-06** |
| SM35:1 | 1.16±0.27 | 0.63±0.15 | 0.97±0.22 | **7.3E-23** | **4.9E-04** | **2.2E-10** |
| SM36:1 | 6.67±0.85 | 7.38±0.83 | 8.65±1.23 | **7.7E-04** | **1.4E-16** | **5.2E-08** |
| SM36:2 | 2.80±0.45 | 1.88±0.25 | 3.16±0.60 | **5.0E-19** | **8.2E-04** | **1.8E-25** |
| SM38:1 | 8.90±1.84 | 10.73±1.94 | 7.86±1.36 | **1.8E-06** | 2.0E-02 | **4.6E-11** |
| SM38:2 | 3.88±0.75 | 4.00±0.68 | 3.08±0.70 | 1.0E+00 | **2.0E-06** | **9.8E-08** |
| SM38:3 | 0.18±0.07 | 0.17±0.05 | 0.12±0.04 | 3.8E-01 | **2.7E-06** | **1.2E-03** |
| SM39:1 | 2.67±0.74 | 3.18±0.74 | 1.71±0.70 | **1.7E-03** | **2.6E-08** | **2.5E-15** |
| SM40:2 | 9.28±1.95 | 9.87±1.90 | 8.53±1.70 | 3.4E-01 | 1.9E-01 | 4.4E-03 |
| SM40:4 | 0.008±0.003 | 0.008±0.003 | 0.012±0.003 | 1.0E+00 | **9.0E-08** | **4.2E-09** |
| SM40:5 | 0.13±0.08 | 0.10±0.05 | 0.13±0.06 | 3.4E-02 | 1.0E+00 | 6.6E-02 |
| SM42:1 | 7.42±1.62 | 5.98±1.30 | 6.26±1.25 | **3.4E-06** | **7.1E-04** | 1.0E+00 |
| SM42:2 | 13.61±2.40 | 13.08±2.17 | 15.24±2.68 | 8.0E-01 | 5.5E-03 | **2.3E-04** |
| SM42:6 | 0.30±0.24 | 0.31±0.19 | 0.41±0.21 | 1.0E+00 | 8.1E-02 | 1.2E-01 |
| SM37:2 | 0.07±0.04 | 0.08±0.04 | 0.06±0.04 | 8.2E-01 | 9.0E-01 | 1.4E-01 |
